# AGING INDUCES NLRP3 INFLAMMASOME DEPENDENT ADIPOSE B CELL EXPANSION TO IMPAIR METABOLIC HOMEOSTASIS

**DOI:** 10.1093/geroni/igz038.397

**Published:** 2019-11-08

**Authors:** Christina Camell, Vishwa Deep Dixit

**Affiliations:** Yale University, New Haven, Connecticut, United States

## Abstract

Visceral adiposity in elderly is associated with alterations in adipose tissue immune cells leading to inflammation and metabolic dysfunction. The Nlrp3 inflammasome is a critical regulator of macrophage activation, inflammation, and immunometabolism in visceral adipose tissue during aging; however, the potential contribution of adipose tissue B cells is unexplored. Here, we show that aging expands adipose-resident B cells and fat-associated lymphoid clusters (FALCs) in visceral white adipose tissue of female mice. Adipose tissue B cells exhibit a memory-like B cell profile similar to the phenotype of aged B cells that are increased in spleen of old mice. Mechanistically, the age-induced FALC formation and adipose B cell expansion, but not B cell transcriptional program, is dependent on the Nlrp3 inflammasome. Furthermore, B cell depletion in aged mice improves insulin sensitivity and metabolic capacity of adipose tissue. These data reveal that inhibiting Nlrp3-dependent B cell accumulation can be targeted to reverse metabolic impairment in aging adipose tissue.

